# The Relationship of Forgiveness and Values with Meaning in Life of Polish Students

**DOI:** 10.1007/s10943-019-00860-4

**Published:** 2019-06-17

**Authors:** Stanisław Głaz

**Affiliations:** Jesuit University Ignatianum in Krakowie, ul. Kopernika 26, 31-501 Kraków, Poland

**Keywords:** Terminal values, Forgiveness, Meaning in life, Process, Polish students

## Abstract

The purpose of this article was to show the relationship of terminal values and forgiveness with meaning in life in Polish students who consider themselves faithful and practicing. The study involved youth studying at the Jesuit University Ignatianum in Krakow. It was carried out among 368 students. The age of the participants ranged between 19 and 23. Three tools were applied: Rokeach Value Survey, Forgiveness Scale by Toussaint, and the Purpose in Life Test of Crumbaugh and Maholick. The analysis of the results obtained proves that terminal values and forgiveness have a statistically significant relationship with meaning in the life of the studying youth.

## Introduction

In recent years, there has been a great interest among scientists in the issue of forgiveness (Exline et al. 2003; Charzyńska and Heszen 2013), values (Głaz 2014; Krok 2015a) and meaning in life (Popielski 2000). Some researchers showed the benefits of individual life and social issues that result from fulfilling the sense of life and values, as well as from forgiving (Worthington 2006; Toussaint et al. 2012; van Tongeren et al. 2014). This article seeks to show the relationship that exists between the terminal values that are associated with the existential purpose of man, forgiveness, and also meaning in life of Polish students who consider themselves believers and practitioners.

### Forgiveness

In human life, there are such phenomena as forgiveness and reconciliation. Both are connected with guilt and harm. They concern the wrongdoer and the harmed person, because forgiveness is a matter for the person and is always possible, while reconciliation is not always possible, and depends on the attitude of the other person. They are associated with a more or less severely harmed person—mentally, spiritually, and even physically (Macaskill 2005). Sometimes researchers use these terms interchangeably, which is not justified. Forgiveness is an internal experience. It means the internal decision of the individual, forgiveness of the harm suffered, and the decision that will change the situation and the relationship between the wrongdoer and the harmed (Olbrycht 2016). Psychologists believe that forgiveness is a psychological construct. It concerns the change in attitude towards the person who caused harm, what manifests itself in thinking, feeling, behaviour. All developed concepts of forgiveness have some basic points, and they deal with the response to the harm being done. Forgiveness definitions have included behavioural (Enright and Coyle 1998), affective (Jaworski 2016), cognitive (Kaleta and Mróz 2016), and motivational (McCullough et al. 2000) components. For a believer, forgiveness is an expression of God’s love and a response to the plea of a man who is contrite for committed iniquities. It is often understood interchangeably with the remission of sins, which belongs only to God. There is a connection with the sacrament of confession, called the sacrament of penance and reconciliation. God’s forgiveness helps the believer to forgive to self, and forgive to others (Jaworski 2016). The beginning of forgiveness takes place when the process of forgiving is followed not only by the disappearance of negative thoughts, feelings of revenge, and hatred against the perpetrator of harm, but also when positive thoughts and feelings about the perpetrator appear (Worthington and Di Blasio 1990). Reconciliation, on the other hand, requires contact with another person, in a religious dimension with God. Forgiveness precedes reconciliation, and it is also a condition of unity and agreement between people. Reconciliation is built on a mutual exchange of forgiveness, that is, when the offering of forgiveness is combined with its acceptance, regardless of the magnitude of fault on both sides. In reconciliation, it is necessary to include parties involved in conflict, the one who has hurt, and the one who has been harmed (Worthington 2006). Reconciliation, like forgiveness, is a process; not infrequently, it takes a long time, and a great patience of both sides is necessary. Reconciliation should also be combined with the readiness to repair the harm done, as much as it is possible. The first step in the process of reconciliation can be done not by the one who is more at fault, but by the one who has more mental and spiritual strength (Exline et al. 2003).

On the basis of psychology, we encounter many definitions of forgiveness. In many respects they differ from each other, but also have much in common (Gorsuch and Hao 1993; McCullough et al. 2000). Most of them were formulated as part of social and developmental psychology as well as pastoral psychology, especially in the context of Christian religion (Wolicki 2006; Zarzycka 2016). Researchers in their theories of forgiveness most often point to one of the important aspects of this issue, for example: the aspect of the individual’s motivation for forgiveness (McCullough et al. 1998), the emotional element (Rye et al. 2001), the personal aspect (Wohl et al. 2008), the pro-social aspect (Hook et al. 2012), or religious aspect (Toussaint et al. 2001). Some scientists, in defining forgiveness, more stress the act of the will of a man (Drinnon and Jones 2009), and others attach greater importance to the process of forgiveness (Jaworski 2016). Forgiveness is an act of will, a decision regarding the relationship and conduct in relation to the harm suffered, as well as to the wrongdoer. It is a conscious, responsible, and free act. The process of forgiveness, on the other hand, opens the person to readiness for reconciliation, which deals with overcoming bitterness and promotes the reconstruction and development of correct interpersonal relations (Jaworski 2016). Forgiveness can also be treated in terms of an attribute (that is predisposition to forgiveness) or as the current state—from psychological point of view. Forgiveness as predisposition is a relatively constant positive trait, a tendency to forgive wrongs experienced at different times and different situations. And forgiveness understood in the category of a state is a response of a man to a specific situation that occurred in his life (Mauger et al. 1992).

Psychologists McCullough and Worthington (1994) believe that the known theories of forgiveness on the basis of psychology can be presented in four different groups. The first group includes forgiveness models based on classical psychological theories, which arose on the basis of psychodynamic theory and cognitive theories, in relation to the object, as well as on the basis of personal constructs. Forgiveness would concern the violation of internalized principles in the past, intrapsychic agreements, and personal norms. Forgiveness is one of the most central of virtues. So central that is linked with salvation. Mature forgiveness does not involve the elimination of negative feeling towards others or oneself but the integration of negative and positive self-object representation and their connected affect (Kelly 1955; Gartner 1988). The second group are the processual theories of forgiveness. They refer to the sequence of sentences and psychological phenomena of an intrapersonal or interpersonal nature that take place during the process of forgiveness (Benson 1992; Enright and Coyle 1998; Wohl et al. 2008). In models of interpersonal forgiveness, both the activity of the person who suffered harm and the activity of the wrongdoer are taken into account. In contrast, intrapersonal models refer to the process of forgiveness, which includes the cognitive, emotional, and behavioural sphere of the forgiver, regardless of the behaviour of the perpetrator after harm. The third category of forgiveness includes models based on the concept of human moral development according to Kohlberg (1976). They suggest that the individual during the development process increases his or her cognitive abilities with age, becomes more open and able to accept the other person’s perspective, empathizes more with the weakness, deficiencies, and difficult life situation, and is therefore more able to understand and accept a person from whom he has suffered in the past (Enright 1991). The fourth group of models are typologies of forgiveness, studies based on the characteristics that differentiate them. Researchers suggest that there is a type of forgiveness without understanding, accompanied by anxiety and fear; the type of instrumental forgiveness which is an immediate means of achieving a given goal through contempt and the type of internal forgiveness that is characterized by a change in attitudes and feelings towards the perpetrator of the wrong. According to these researchers, on the basis of behavioural and emotional changes in behaviour towards the wrongdoer, they mention the impartial, limited, and full type of forgiveness (McCullough and Worthington 1994). Based on the aforementioned theoretical concepts of forgiveness, scientists have developed several tools to measure this phenomenon, its structure, and effects (McCullough and Worthington 1994; Kaleta and Mróz 2016; Noworol 2016).

### Meaning in Life and Values

According to some researchers (Frankl 1987; Popielski 2000), meaning in life and values are two inseparable components of human life. Creative human involvement (Kinget 1975) is connected with the will to make sense, motivates to discover the meaning in life and realize values (Frankl 1992). Meaning in life is understood as one of the needs (Maslow 1968). It involves the realization of a person in relation to society (Adler 1964), in updating individual goals and realizing religious values (Bühler 1962); it is carried out while making important life decisions (May 1981; Leontiev 2003). Wong (1998) defined meaning as an individually constructed, culturally based cognitive system that influences an individual’s choice of activities and specific goals and endows life with a sense of purpose, personal worth, and fulfilment. Steger and Frazier’s (2005) definition of the meaning in life is the sense made of and significance felt regarding the nature of one’s being and existence. The author proposed a model of the meaning in life, within which he distinguished two of its dimensions: the presence of the meaning in life and the search for the meaning in life. He draws attention to the need to distinguish the time perspective while studying the dimension of the presence of meaning in life and its search. The first of these, the presence, expresses the conviction that life has a clear sense and purpose and that man is able to properly assess his current level of meaning in life and recognize the factors responsible for its formation. The second dimension, searching, represents a state in which man does not have a satisfying sense of the meaning in life and seeks to discover goals and values that can make his life more meaningful (Steger 2011). According to this concept, people may have the meaning in life or strive to achieve it. Both of these time dimensions have a slightly different meaning for human life, but they are complementary (Kossakowska et al. 2013). Frankl proposes the existential view and understanding of the meaning in life. It is associated with adversities and everyday challenges. According to Frankl (2000), man is not a being driven by his impulses but is attracted by life goals and values. The meaning lies in life itself: it is the basic need of needs. Man realizes the meaning in life when he fulfils himself as a person, when he is focused on achieving goals and values (Absolut, salvation, love of the other, beauty). Meaning in life as a psychological construct emerged in part as a reaction to World War I and II. Frankl’s experiences as a prisoner in the concentration camps tested and validated his theory. While imprisoned, Frankl observed the differences amongst the prisoners who were able to maintain or hold onto some meaning in their lives compared to those who lost meaning while imprisoned. According to Frankl (1992), the sense of life is connected with the sense of the meaning in life (Frankl 1987). The sense concerns the subjective belief that meaning in life and values are realized. The feeling of the sense of life contains several components. The intellectual component refers to the knowledge of man and his personal goals, life history. An emotional-volitional element is associated with the ability to experience oneself, to respond to values. The volitional-aspirational element is connected with the man’s ability to make choices and existential-action concerns the belief in realizing meaning in life.

Meaning in life is related to values. Schwartz (2011) defined values as trans-situational, desirable goals, varying in importance, that serve as guiding principles in people lives. According to Rokeach, the value concept is a constant belief that a specific mode of conduct or end (ultimate) state of existence is personally and socially superior to an opposite or converse mode of conduct or end state of existence. Rokeach (1969) considers value to be a type of belief that is centrally located within one’s total system of believes regulating behaviours. Rokeach distinguished two kinds of values: instrumental and terminal. Terminal values can be of personal and of social character and define the end state of human desires and aspirations (such as personal freedom, salvation). Terminal values refer to desirable end-states of existence. These are the goals that a person would like to achieve during his or her lifetime. Instrumental values are of moral character and denote competency and are seen as desirable modes of conduct (e.g. helpfulness, self-control). The terminal values denote aims that people set, whereas the instrumental values are modes of conduct thanks to which those aims can be achieved. Particular values exist within the system of the elderly and indicate the hierarchy of values. Many researchers (Rokeach 1973; Popielski 2000) suggest that values are attractive, they fascinate people, they demand realization, they are manifested in the path of human life activity. The values not only fulfil psychological functions, i.e. they outline the principles of individual and social life (Popielski 2000), define the principles of interpersonal communication (Opoczyńska 1995), but also perform a religious function, namely—orientate the human being to the transcendent world (God, beauty). They are connected not only with his intellectual and emotional sphere, but also with his existential dimension. The values have a universal function of the regulator in the process of human development (Rokeach 1969). Lack of meaning in life is often associated with the lack of implementation of significant values in life, which manifests itself in many negative aspects of individual and social life, sometimes in the form of existential neurosis or noogenic neurosis (Frankl 2000).

### The Problem and Objective of the Research

Frankl (1992) understands meaning of life as a life purpose and a life task. He claims that it is the idea that has become the value of a very high order for a human; it sets the basic orientation of his or her life and assumes a certain way of experiencing life itself. The purpose and sense of achieving this goal is connected with meaning in life (Baumeister 1991), as well as the realization of values (Popielski 2000). Research shows a number of personal and social benefits resulting from realizing the sense of one’s life and values, as well as the blessing resulting from forgiving the wrongs committed. In addition, it displays the damage resulting from the non-alignment of meaning in life and value and because of the lack of forgiveness to oneself and others (Worthington and Di Blasio 1990).

Research by Salikhova (2015) shows that the American students are more concerned about freedom, while Russian—about friendship. The results obtained in the Rokeach Value Survey show that Polish students with a high level of empathy amongst terminal values prefer mostly wisdom, pleasure, and family security. Similarly, students with a low level of empathy prefer pleasure and freedom as well as family security. Whereas in the group of people with a high level of empathy the value—equality—contributes more to explain the variance of religious experience of God’s absence, and in group of people with a low level of empathy, it is the value of salvation (Głaz 2014). Seminary students of philosophy pointed to four terminal values: inner harmony, wisdom, salvation, and freedom, whereas students of physics chose pleasure, wisdom, and a world at peace and family security. Only one value—wisdom—out of the four most preferred values was pointed to by both seminary students of philosophy and students of physics. In the group of seminary students of philosophy, from amongst the four most preferred terminal values, two have a significant relation with the experience of God’s presence and God’s absence, whereas in the group of students of physics only one of them has a significant relation with the experience of God’s absence (Głaz 2016). The results obtained in the Rokeach Value Survey concerning instrumental values demonstrate that out of the 18 values, students of pedagogy highly ranked broad-mindedness, ambitiousness, helpfulness, and responsibility, whereas students of philosophy above all favoured instrumental values such as responsibility, imaginativeness, logicality, and capability (Głaz 2015). The study on persons randomly recruited in southern Poland show that aesthetic, truth and moral values were positively associated with task-oriented coping, while hedonic and vital values were positively linked to emotion-oriented and avoidance-oriented coping styles. With regard to religious coping styles, vital, aesthetic, truth and moral values were positively associated with positive coping. Negative coping was positively related to hedonic values, but negatively related to sacred values. The centrality of religiosity dimensions was positively related to emotion-oriented coping, avoidance-oriented coping, social diversion and positive religious coping (Krok 2015a).

Steger and Frazier (2005) conducted two studies among students and found that meaning in life mediated the relation between religiousness and life satisfaction as well as the relationship between religious behaviours and well-being. Authors concluded that religious individuals might feel greater well-being because they derive meaning in life from their religious feelings and activities. High level of religiosity is related to lower feeling of meaninglessness in life and average scores on the Purpose in Life Test scale. Research conducted among Polish students reveal a positive correlation between the results obtained in The Personal Meaning Profile: achievement, relationship, religion, self-transcendence, self-acceptance, intimacy, and fair treatment, and the result obtained in The Satisfaction With Life Scale. Four dimensions of personal meaning: achievement, relationship, self-acceptance, and intimacy were negatively associated with the negative affect—the result obtained in The Positive and Negative Affect Schedule (Krok 2018). It was shown that results obtained in Brief RCOPE, concerning positive religious coping positively correlated with results obtained in the Meaning in Life Questionnaire with presence of meaning in life, and search of meaning in life. Negative religious coping was negatively correlated with presence, but not with search. However, negative correlations were found between negative religious coping and results obtained in the Psychological Well-Being Scale: autonomy, environmental mastery, personal growth, positive relations with others, and self-acceptance (Krok 2015b). At the same time, low level of religiosity among participants with low self-concept clarity is related to the lowest scores on the Purpose in Life Test scale (Błażek and Besta 2012). Persons pursuing the Christian or the Oriental form of meditation revealed no significant differences with regard to the intensity of the sense of meaning in life (Kulik and Szewczyk 2002).

Research conducted among the Polish community by Charzyńska and Heszen (2013) prove that there is a positive correlation between the three factors of forgiveness according to Toussaint concept: forgiveness to self, forgiveness to others, and a sense of forgiveness by God. There are numerous health benefits when engaging in the process of forgiveness; examples of some of the benefits are a reduction of negative thought processes and emotions (Worthington 2006). The benefits of forgiveness also extend to an individual’s ability to maintain relationships with others by way of the reparation from conflict caused by the effects of negative thought processes and emotions (Gordon and Baucom 1998). Maltby et al. (2001) showed in the group of undergraduate students who failed to forgive others and/or failed to forgive themselves had higher depression scores compared to those who could forgive themselves and/or others.

Karseboom research (2016) conducted among students at a Canadian college suggests higher level of forgiveness towards others than towards the situation, towards the self, and overall forgiveness. The study found that there is a positive relationship between meaning in life measured by Meaning in Life Questionnaire and dispositional forgiveness measured by the Heartland Forgiveness Scale, but no relationship between meaning in life and dispositional forgiveness of others, as well as a positive relationship between meaning in life and dispositional forgiveness of situations, and finally positive relationship between meaning in life and overall dispositional forgiveness. Studies by van Tongeren et al. (2014) revealed that dispositional forgiveness and the degree of forgiveness following an offense were positively related to meaning in life. Participants who regularly forgave their partner reported increased meaning in life over time. In addition, forgiveness helped recover the lost meaning among those participants reporting more frequent partner offenses.

As mentioned in the opinion of many researchers, the psychological and spiritual health of man and his development depend on realizing the meaning in life (Frankl 1987; Leontiev 2003), realizing values (Popielski 2000), and the ability to forgive oneself and others as well as the sense of forgiveness by God (Worthington and Di Blasio 1990; Toussaint et al. 2001). Psychological and pastoral counselling as well as some studies confirm that people who forgive others cannot always forgive themselves (Grün 1997; Toussaint et al. 2012), and sometimes believers feel that their faults have not been forgiven by God. In addition, people prefer different values, and they display a different degree of sense of life. However, the results of research on value, forgiveness, and sense of life are not explicit. They are differentiated by the age of the respondents, culture, belonging to a religious group, temperamental, and characterological characteristics, as well as the operationalization of the issue accepted by the researcher. Further research is required in this regard. Christian religion provides norms indicating that forgiveness is good and that good religious people should forgive, and it would make sense that religious people have more positive attitudes towards being forgiving and should want to be more forgiving. Religion shows that the meaning of human life is fulfilled when a person realizes such values in his life as love, forgiveness, responsibility, and when he perfects and cares for his own salvation. In addition, research has indicated (McCullough and Worthington 1994; Popielski 2000) that people with a high level of religiousness are more able to forgive and have a greater sense of God’s forgiveness and value more mature love and salvation than people with low religiousness.

The aim of the paper is to show how much terminal values and forgiveness have beneficial impact on meaning in life in the life of Polish students who declare themselves believers and practitioners. Therefore, it was decided to show the relationship of the terminal values in the sense of Rokeach using Rokeach Values Survey (RVS) and the presence of meaning in life as assessed by the Purpose in Life Test (PIL) and with forgiveness as assessed by The Scale of Forgiveness (SF) in the life of students. Personal life and social behaviours of Poles are strongly embedded in the religion of Roman Catholic denomination, which calls for forgiveness, for the love of even enemies, and encourages the development of religious life. According to research, despite the departure of young people in the period of socio-economic transformation from some values to others, some basic values, as well as religion, still fulfil an important regulatory and motivational function in the youths’ personal and social life (Popielski 2000; Głaz 2014). It is suggested that the studying youth considering themselves to be practicing believers, mostly value of 18 terminal the following values: salvation (saved, eternal live), wisdom (a mature understanding life), and true friendship (close companionship), and also has high level of forgiveness to self, forgiveness to others, and a sense of God’s forgiveness. It is postulated that the terminal values mostly valued by students that, according to Rokeach (1969) and Popielski (2000), perform an important regulatory and motivational function in meaning in life, perform also functions in the process of forgiveness: forgiveness to self, forgiveness to others, and a sense of God’s forgiveness. It is expected that cognitive, emotional, behavioural, and religious elements of forgiveness: forgiveness to self, forgiveness to others, and a sense of God’s forgiveness have significantly and positive correlated with meaning in life of believers students. In addition, it is suggested that there is a significant relationship between terminal values and meaning in life in the lives of adolescents-believers of different types of forgiveness, and it is also postulated that the three dimensions of forgiveness fulfil an important intermediate function between the most preferred terminal values and meaning in life, which is related to the realization of life goals and oneself as a person. This issue is the essence of this article and will be used to formulate research hypotheses.

### Hypotheses

1. The most valued terminal values have a significant relationship with meaning in life in students’ lives
2. Forgiveness to self, forgiveness to others, and a sense of forgiveness by God have a significant relationship with meaning in life in the lives of students.
3. There is a significant relationship between terminal values and meaning in life in the lives of young people with different types of forgiveness.
4. Forgiveness has an important mediating role between three most valued terminal values and meaning in life in the lives of students.

## Methods

### Procedure and Participants

The research was carried out after the academic classes among those students who voluntarily expressed their willingness to participate in this kind of event. The first section of the questionnaire was aimed at obtaining basic demographic information in relation to participants’ age and gender. Questions considered the place of birth, membership in a religious group, as well as religious commitment. In the second section of the questionnaire, a variety of standardized questionnaires were used to measure participants’ preference of values terminal, level of meaning in life, and level of forgiveness. The research was carried out in Krakow. It included students (full-time and part-time) of a private university (Jesuit University Ignatianum). All respondents were born in Poland and grew up in a Catholic family. All the students declared belonging to the Roman Catholic Church. They consider themselves believers and practitioners. They take part in the Sunday Eucharist, they pray, they use the sacraments. Students asked how often they use religious practices answered in the following way: 23.0% of the youth claimed that they practice very often, 42.4% often, 21.3% rarely, and 13.3% very rarely. The age of the participants ranged between 19 and 23 (*M* = 20.16; SD = 12.19). The result of 368 correctly completed sets of questionnaires were then analysed.

### Measures

In order to solve the problem in this work, the following research tools were used: Rokeach Values Survey (RVS), the Purpose in Life Test (PIL) of Crumbaugh and Maholick, and The Scale of Forgiveness (SF) by Toussaint.

#### Rokeach Values Survey (RVS)

The scale contains eighteen terminal values and eighteen instrumental values (Rokeach 1969, 1973). Only terminal values are included in this article. These values concern the existence of a man and his life goals. The scale suggests that the values should be ordered from the most important value in human life to the least important value. These are—(1) national security, (2) family security, (3) mature love, (4) a comfortable life, (5) wisdom, (6) a sense of accomplishment, (7) self-respect, (8) a word at peace, (9) true friendship, (10) pleasure, (11) inner harmony, (12) equality, (13) happiness, (14) a world of beauty, (15) social recognition, (16) freedom, (17) salvation, (18) an exciting life. Rokeach estimated the stability of each value (test reliability) by the test–retest method (*N* = 250), and the scores for terminal values were coefficients ranging from .51 to .88. The scale was adapted to Polish condition by Brzozowski (1986). The rank correlation coefficient between the Polish and American versions of the scale of terminal values is .89.

#### The Purpose in Life Test (PIL) of Crumbaugh and Maholick

The test measures the degree to which the subject experiences a sense of meaning in life (Crumbaugh and Maholick 1964). It was created based on a theoretical understanding of the meaning in life according to Frankl (1992), who understands meaning in life as a purpose and a task. Meaning in life is considered as a binary construct having both existential and positive psychological characteristics. All items in the scale are rated by a 7-point scale using the response categories of absolutely untrue (1), mostly true (2), somewhat untrue (3), can’t say true or false (4), somewhat true (5), mostly true (6) or absolutely true (7). The task of respondent is to choose an opinion on the seven-point Likert-type scale expressing to what extent he or she agrees o disagrees with a given statement. The high overall score of the scale indicates a high level of meaning in life, and a low score shows a low level of meaning in life. The test was adapted to Polish condition by Popielski (1987). Cronbach’s alpha coefficient of internal consistency for consecutive items ranges from .88 to .92.

#### The Scale of Forgiveness (SF) by Toussaint

It is a multidimensional tool designed to measure forgiveness, which also takes into account dimensions such as cognitive, emotional, and behavioural as well as religious forgiveness (Toussaint et al. 2001). The adaptation of the scale to Polish conditions was made by Charzyńska and Heszen (2013). Each statement on the scale is accompanied by five possibilities of answers. The task of respondent is to choose an opinion on the five-point Likert-type scale expressing to what extent he or she agrees or disagrees with a given statement. The examined uses categories of almost always false of me (1), more often false of me (2), can’t say true or false (3), more often true of me (4) or almost always true of me (5). The high overall score of the scale indicates a high level of forgiveness, and a low score for a low level of forgiveness. Three factors have been taken into account in this work. The first factor concerns self-forgiveness (PA)—involves release of negative affect and self-blame associated with past mistakes, wrongdoings; the second forgiveness to others (PB)—involves forgiving another for some harm done; and third is the forgiveness of God (PC)—refer to the believe or perception that one’s transgressions are forgiven by the divine. The reliability of the scale evaluated with Cronbach’s *α* is: .65 ≤ *α* ≤ .91, and the absolute stability ratio of the tool is: for the first factor *p* = 0.70, for the second *p* = 0.76, and for the third *p* = 0.81.

### Statistical Analysis

The analysis of variance (ANOVA) was applied. The results analysis was carried out on the basis of mean values (*M*) and standard deviations (SD). For variables (terminal values) expressed by means of ranks a nonparametric test (*U* Mann-Whitney test) was used. In order to determine the strength of the relationship and its character between the variables taken in this work, Pearson’s correlation coefficient *r* was calculated and the multiple regression analysis procedure was applied. In order to show more complex causal relationships between variables, the technique of structural equations was used, and Cluster Analysis was used to group people with different types of forgiveness.

## Results

The analysis of the obtained results allowed the verification of the hypotheses concerning the relation between forgiveness and terminal values and meaning in life in the lives of students who consider themselves believers and practitioners.

### The Level of Forgiveness and Meaning in Life and Pearson’s Correlation. Ranking Distribution of Terminal Values in the Lives of Student (Table 1)

The results obtained in the Rokeach Value Survey (RVS) concerning terminal values show that students respect most values: (1) a comfortable life (a prosperous life), (2) family security (taking care of loved ones), (3) equality (brotherhood, equal opportunity for all). The results obtained in Forgiveness Scale (FS) show that students obtained a high score in the forgiveness factor for others (PB) (*M* = 4.0; SD = 0.975), and the average in self-forgiveness factor (PA) (*M* = 3.6; SD = 0.530) and in the factor of God’s forgiveness (PC) (*M* = 3.4; SD = 0.823). Forgiveness for others (PB) correlates positively with self-forgiveness (PA) (*r* = 0.24; *p* < 0.01). However, the overall result obtained in the Purpose in Life Test (PIL) regarding meaning in life (*M* = 4.9; SD = 0.856) is high. It correlates positively with all three components of forgiveness: self-forgiveness (PA) (*r* = 0.34; *p* < 0.01), forgiveness for others (PB) (*r* = 0.24; *p* < 0.01), a sense of God forgiveness (PC) (*r* = 0.17; *p* < 0.05) (Table 1).

**Table 1:** Average (*M*), standard deviation (SD), and the results of the Pearson’s correlation for components of forgiveness: forgiveness of self (PA), forgiveness of others (PB), a sense of God forgiveness (PC), and meaning in life (PIL). Three most respected terminal values

Variables	*M*	SD	PA	PB	PC	PIL	Three most respected values
PA	3.6	0.603	1.00	–	–	–	
PB	4.0	0.975	0.24*	1.00	–	–	A comfortable life
PC	3.4	0.823	− 0.01	0.07	1.00	–	Family security
PIL	4.9	0.856	0.34**	0.24**	0.17*	1.00	Equality

**p < 0.01; *p < 0.05

### Relation of Terminal Values and Forgiveness with Meaning in Life in the Life of Students (Tables 2, 3)

It was decided to show which of the terminal values and components of forgiveness: forgiveness to self (PA), forgiveness to others (PB), and a sense of forgiveness by God (PC) have a statistically significant relationship with meaning in life and how they explain the variance of meaning in life in students’ lives. For this purpose, a multiple regression analysis procedure was applied. The independent variable is the terminal values obtained in Rokeach Value Survey (RVS) and in The Scale of Forgiveness (SF), while the dependent variable is the result obtained in the Purpose in Life Test (PIL). The results of multiple regression analyses are shown in Tables 2 and 3.

**Table 2:** Independent variable relating to terminal values explaining the variance of meaning in life in the lives of young people

Regression summary for dependent variable: meaning in life (PIL) *R* = 0.33; *R*^2^ = 0.11; *F*(18,349) = 2.47; *p* = 0.0008				
Variable	*β*	*b*	*t*	*p* value
Intercept		4.93	4.72	< 0.0001
A comfortable life	0.18	0.04	2.77	0.006

**Table 3:** Independent variables relating to forgiveness to self (PA), forgiveness to others (PB), forgiveness by God (PC) explaining the variance of meaning in life of young people

Regression summary for dependent variable: meaning in life (PIL) *R* = 0.40; *R*^2^ = 0.16; *F*(3364) = 23.78; *p* < 0.0001				
Variable	*β*	*b*	*t*	*p* value
Intercept		3.59	10.57	< 0.0001
PA	0.28	0.42	5.73	< 0.0001
PB	0.18	0.17	3.89	0.0002
PC	0.17	0.19	3.61	0.0003

Only one terminal value out of 18 values, and it is a comfortable life, has a statistically significant and positive relationship with meaning in life. It explains 11% of the variance of meaning in life (Table 2).

All three components of forgiveness: forgiveness to self (PA), forgiveness of others (PB), and a sense of forgiveness by God (PC) have a statistically significant relationship with meaning in life. Their relationship is positive. They explain 16% of the variance of meaning in life (Table 3).

### Types of Forgiveness and the Relation of Terminal Values with Meaning in Life in Groups of People with Different Types of Forgiveness (Fig. 1)

**Fig. 1:**
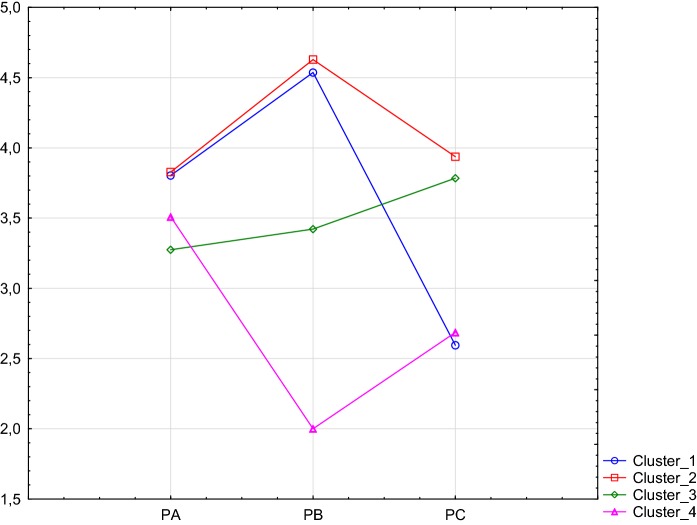
Graphical distribution of results for four types of forgiveness

In order to determine the types of forgiveness of young people studying, the results obtained on The Scale of Forgiveness (SF) were subjected to cluster analysis, where the *K*-means method was used. In this way, four homogeneous groups were created with different numbers of people (Fig. 1). Statistically significant differences between clusters in specific factors were determined using the Wilks test (*F* = 145, *df* 9; *p* < 0.0001).

The first cluster consists of 95 people. They are young people with high level of forgiveness to others (PB) (*M* = 4.5; SD = 0.511), average self-forgiveness (PA) (*M* = 3.8; SD = 0.564) and a low result of God’s forgiveness (PC) (*M* = 2.6; SD = 0.522). This type of forgiveness can be called humanistic. The second cluster included 136 people. They are youth with a high score of forgiveness to others (PB) (*M* = 4.6; SD = 0.408) and the average result in the other two factors, i.e. self-forgiveness (PA) (*M* = 3.8; SD = 0.667) and a sense of forgiveness by God (PC) (*M* = 3.9; SD = 0.465). This type of forgiveness can be called humanistic-religious. The third cluster consists of 102 people. These are young people with average results in all three factors: self-forgiveness (PA) (*M* = 3.3; SD = 0.546), forgiveness to others (PB) (*M* = 3.4; SD = 0.481), and a sense of God’s forgiveness (PC) (*M* = 3.7; SD = 0.574). This type of forgiveness can be called demented. The last fourth cluster included 35 people. They are young people with an average result of self-forgiveness (PA) (*M* = 3.5; SD = 0.716), and a very low forgiveness to others (PB) *(M* = 2.0; SD = 0.831), as well as a sense of God forgiveness (PC) (*M* = 2.6; SD = 0.891). This type of forgiveness can be called non-religious.

Next, it was decided to show which of the terminal values have a statistically significant relationship with the meaning of life in groups of young people with different types of forgiveness and to what extent they explain the variances of meaning in life. The obtained results of regression analysis are presented in Table 4.

**Table 4:** Relationship between independent variables relating to terminal values with meaning in live in groups of young people with different types of forgiveness

Youth of the humanistic-religious type of forgiveness Regression summary for dependent variable: meaning in life (PIL) *R* = 0.24; *R*^2^ = 0.18; *F*(18,117) = 2.14; *p* = 0.008				
Variable	*β*	*b*	*t*	*p* value
Intercept		7.70	3.40	0.0017
A world of beauty	− 0.27	− 0.04	− 2.17	0.032
Inner harmony	− 0.24	− 0.04	− 2.16	0.033
Freedom	− 0.27	− 0.06	− 2.20	0.029
A comfortable life	0.23	0.11	2.00	0.048

Youth with a demented type of forgiveness Regression summary for dependent variable: meaning in life (PIL) *R* = 0.56; *R*^2^ = 0.32; *F*(18,83) = 2,15; *p* = 0.011				
Variables	*β*	*b*	*t*	*p* value
Intercept		5.70	2.31	0.024
An exciting life	− 0.31	− 0.07	− 2.17	0.032
A comfortable life	0.28	0.06	2.01	0.047

In the group of youth about the humanistic-religious type of forgiveness (Table 4), four terminal values: a world of beauty, inner harmony, freedom, and a comfortable life have a statistically significant relationship with meaning in life and explain 18% of the variance. The relationship between values and meaning in life is negative, except for one value—a comfortable life. Similarly, in the group of adolescents with demented type of forgiveness two qualifying values: an exciting life and a comfortable life have a statistically significant relationship with meaning in life and they explain 32% of the variance. The relationship of the first value is negative and the second is positive with the meaning in life. While in the group of youth with a humanistic type of forgiveness and in the group of youth with a non-religious type of forgiveness, no terminal value has a statistically significant relationship with meaning in life.

### Mediational Function of Forgiving Between Terminal Values and Meaning in Life in the Life of Students (Fig. 2)

**Fig. 2:**
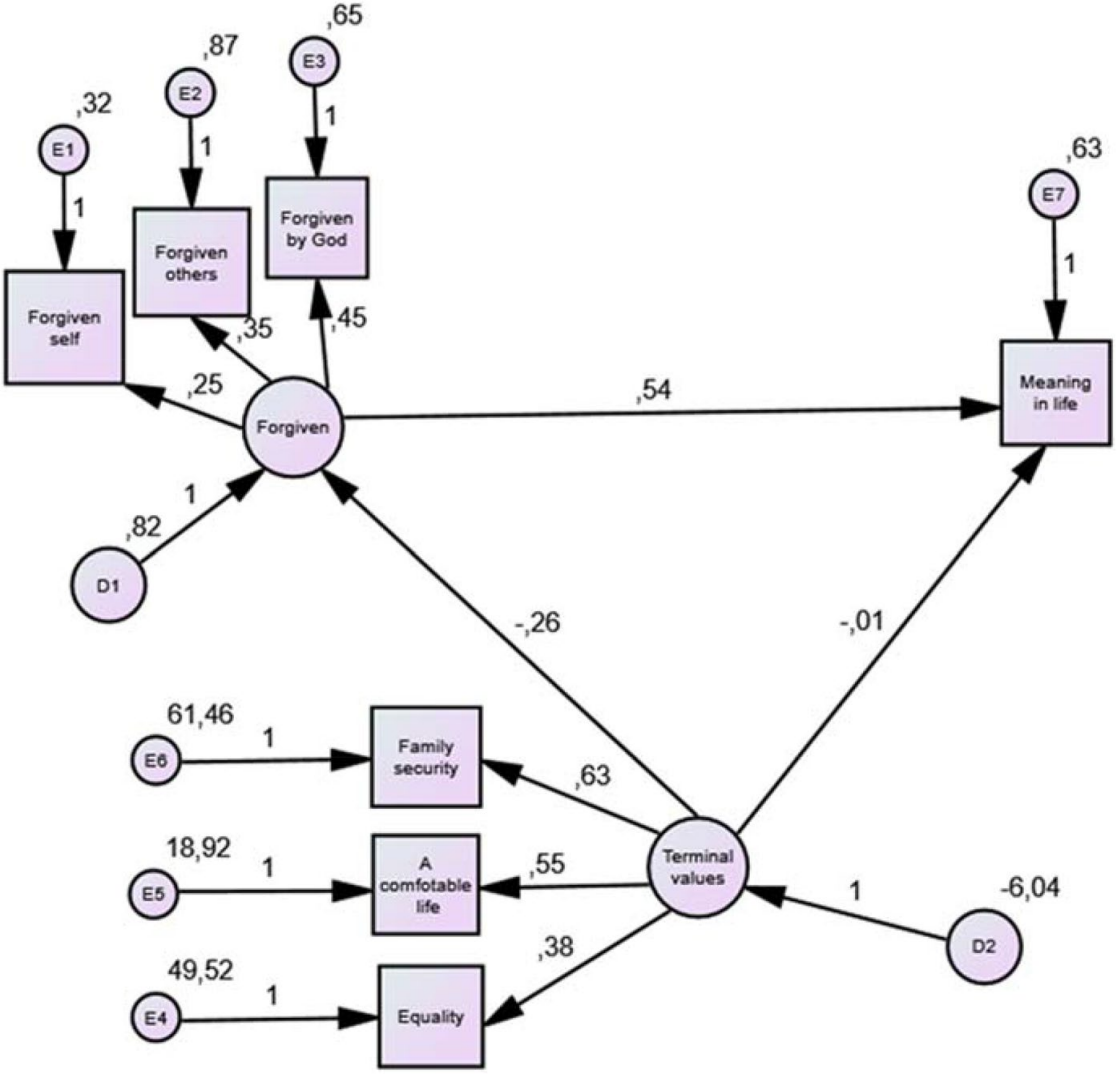
A path diagram showing the relationship between the three most valued values, forgiveness, and meaning in life

According to the fourth hypothesis, it was later decided to examine the postulated causal relations in the variable sets and determine their dependencies. It was assumed that the independent variable is the three most valued terminal values [(1) a comfortable life, (2) family security, (3) equality], the dependent variable—meaning in life, and mediation variable—forgiveness (forgiveness to self, forgiveness of others, a sense of forgiveness by God) (Fig. 2). The analysis was carried out on the data obtained in the Values Survey (RVS), the Purpose in Life Test (PIL), and The Scale of Forgiveness (SF). A model was developed, which was then tested. The quality indicators of the model fit are: RMSEA = 0.101; GFI = 0.931; AGFI = 0.899; *p* < 0.05. The values of the indicators for the tested model proved to be unsatisfactory (RMSEA = 0.101; *p* < 0.05). This suggests that the postulated theoretical model is not completely accurate. However, it was decided to present it. The correlations of variables are presented in the figure, indicating the size of non-standardized path coefficients—effects of interactions—for the relationship between variables.

The obtained results of the analysis show that the strongest non-standardized path coefficient is located between the three components of forgiveness as mediators and meaning in life (*b* = 0.54), while the weakest and non-significant between the three most valued terminal values and the meaning in life (*b* = − 0. 01). There is a significant direct impact of the three terminal values of the most appreciated on the three components of forgiveness (*b* = − 0.26, their relationship is negative). This means that the realization of such terminal values is not conducive to forgiveness. The three components of forgiveness have a significant direct impact as mediators on the meaning of life (*b* = 0.54), which suggests that along with forgiveness the meaning of life increases, and the most valuable values also have a direct impact on meaning in life, but it is not significant (*b* = − 0.01) .

## Discussion of the Results and Conclusion

The purpose of this article was to show the relationship of forgiveness and terminal values with meaning in life in Polish students’ life who declare themselves believers and practitioners. The research hypotheses were verified and several conclusions resulting from the analysis of the issue were pointed out.

The first hypothesis, which postulates that the most valued terminal values have a statistically significant relationship with meaning in life in students’ lives, has been confirmed only partially. Young people indicated the three most-valued values out of 18 terminal values. These are: a comfortable life, family security, and equality. Only one of them—a comfortable life—positively correlates with meaning in life (*r* = 0.18; *p* < 0.05). The same terminal value—a comfortable life—is also found in the regression model and has a significant positive relationship with meaning in life, where it explains 11% of the variance of the meaning of life. It suggests that the more comfortable and prosperous life of young people is, the greater the sense of their own life. The same terminal value—a comfortable life—also has an important relationship in the regression model with meaning in life in the lives of youth about the humanistic-religious type of forgiveness and the demented type of forgiveness.

The second hypothesis, which assumed that forgiveness to self, forgiveness to others and a sense of forgiveness by God have a significant relationship with meaning in life in the life of students, has been confirmed entirety. All three components of forgiveness: forgiveness to self (*r* = 0.34; *p* < 0.01), forgiveness to others (*r* = 0.24; *p* < 0.01) and a sense of forgiveness by God (*r* = 0.17; *p* < 0.05) correlate with meaning in life. It suggests that the greater the ability to forgive oneself and others, and the greater the sense of God’s forgiveness, the greater the sense of meaning in life. In addition, all three components of forgiveness, as predictors, are in the regression model and have a statistically significant relationship with the sense of youth, where they explain 16% of the variance of meaning in life. The relationship of forgiveness to self and to others, as well as a sense of forgiveness by God with meaning in life is positive. It is the more powerful, the greater the ability to forgive oneself and the others and the greater sense of God’s forgiveness, the greater the striving for meaning in life

The third hypothesis, which suggests that there is a significant relationship of the terminal values—as predictors of meaning in life—with meaning in life in the lives of young people with different types of forgiveness, has been confirmed only partially. In the group of people with a humanistic type of forgiveness and a non-religious type of forgiveness, no terminal value has a statistically significant relationship with the sense of youth’s life. In the youth group with the humanistic-religious type of forgiveness, such terminal values as a world of beauty, inner harmony, freedom, and a comfortable life have a statistically significant relationship with meaning in life and explain 18% of the variance. The first three of them have a negative relationship with sense the sense of life, and the last one—a comfortable life—positive. This means that in the lives of young people with a humanistic-religious type of forgiveness, the more comfortable and prosperous life, the greater sense of life, and the greater interest in the beauty of the world, more freedom from inner conflict, more independence, the less sense of life. In addition, in the lives of adolescents with a demented type of forgiveness, the value of a comfortable life—has a positive relationship with meaning in life, and the value an exciting life has a negative relationship with meaning in life, together they explain 32% of the variance. It suggests that in the lives of adolescents with a demented type of forgiveness, the more life excitement and activity, the less sense of life, and the more comfortable and prosperous life, the greater sense of life.

The fourth hypothesis, which postulates that forgiveness plays an important mediating role between the three most valued terminal values and meaning in life in the lives of students, has been confirmed only partially. The obtained indicators show that the model does not fully reflect the theory of Rokeach (1973) and Popielski (2000), which suggests that the most valued terminal values should have a statistically significant relationship with such elements of human life as forgiveness, meaning in life. In this case, the three most valued terminal values: a comfortable life, family security, and equality do not support meaning in life, while meaning in life is aided by forgiveness. That suggests that forgiveness helps by increasing meaning in life when offense is high, and forgiveness may serve an existential function of providing meaning in life.

It is expected that young people who consider themselves to be believers and practitioners were expected to value values such as salvation (saved, eternal live), wisdom (a mature understanding life), and true friendship (close companionship), and a higher level of forgiveness and meaning in life. These components are associated with the Christian religion, which calls for forgiveness of faults and negligence even for enemies, encourages people to love others and God, and also calls to care for their own salvation. Young people now appreciate the most comfortable life, family security and equality, and display an average level of forgiveness and sense of life. This suggests, as other researchers have noted (Jaworski 2016), that the youth in this case probably treat their own religiosity too instrumentally and attach greater importance to acquiring knowledge and graduating from studies than to seek comprehensive development.

What is more, it was expected according to the research of other scientists (Popielski 2000; Krok 2015b) that a greater number of terminal values that have an important regulatory and motivational function in the personal and social life of young people has a significant and strong relationship with the meaning in life and forgiveness in the lives of these students. This assumption has not been confirmed in this case. This may suggest that young people are more interested in gaining knowledge than in discovering the importance of values, may indicate lack of motivation to realize values, and also signal the crisis of values caused by socio-economic changes in the country (Marianski 2001).

Out of the three most-valued terminal values (a comfortable life, family security, equality) only one—a comfortable life—correlates significantly and positively with three components of forgiveness and meaning in life, and this value was included in the regression model explaining its relationship with sense of youth life. This would mean that the stability of life and decent life are conducive to young people studying to realize themselves as a person, their own talents and abilities, and mobilizing them to forgive and realize the meaning in life.

Correlation analysis between the components of forgiveness is as follows: forgiveness to self correlates with forgiveness to others. Their relationship is positive. On the other hand, both components of forgiveness do not really correlate with a sense of God’s forgiveness, which would suggest that forgiving the wrongdoer also brings many benefits to the injured person. In order to forgive oneself and others, it is not necessary to be convinced of God’s forgiveness of wrongs. The lack of a positive relationship between forgiveness to self and forgiveness to others with a sense of God’s forgiveness may suggest that young people do not perceive God as merciful. The result of this research, in this case, was confirmed by previous studies carried out on the Polish population by Charzyńska and Heszen (2013), as well as among other groups of people examined by Mauger et al. (1992).

In addition, all three components of forgiveness significantly correlate with meaning in life. The relationship between forgiveness to self and forgiveness to others, as well as a sense of God’s forgiveness and meaning in life is positive. What would mean, in this case, that the student realizes himself, his own talents and habits, the greater the ability to forgive his own neglect and forgive others their mistakes, and at the same time a greater sense of God’s forgiveness for his faults. All three components of forgiveness, as predictors of meaning in life, are also found in the regression model and explain 16% of the variance of meaning in life. This result was not confirmed by Karseboom’s previous studies (2016), which indicated that forgiveness to others does not correlate significantly with meaning in life. It suggests, in both cases, that the understanding of forgiveness proposed by researchers, which includes the human and religious factors, favours young people to realize themselves as a person, and also enables the realization of the meaning of life (van Tongeren et al. 2014). According to Popielski (2000), this may suggest that forgiveness also plays an important role of self-regulation in the lives of young people.

Analysis of the results shows that young people have obtained an average result of forgiveness to self, forgiveness to others and a sense of forgiveness by God in the scale of forgiveness. Similar results were obtained by Karseboom (2016) among Canadian college students. This suggests that young people can understand the harm suffered and people from whom they have suffered, and is also able to emotionally overwork the harm experienced. In addition, they believe that the mistakes they made were forgiven by God.

The proposed multidimensional model of forgiveness by Toussaint et al. (2001), which covers the cognitive, emotional, behavioural and religious sphere of man indicates that the lack of forgiveness leads to revenge against the wrongdoer, the lack of forgiveness favours self-blaming, a sense of shame, and lack of sense of God’s forgiveness leads to a lack of trust in God’s mercy. On the other hand, the ability to forgive oneself and others leads to better relationships with oneself and others, and also with God, and the sense of God’s forgiveness helps the person understand that immoral acts have been forgiven by God (Grün 1997). The multidimensional approach to forgiveness can be widely used in psychotherapy, and pastoral work. Such an understanding of forgiveness can serve believers as well as non-believers. In the lives of believers and non-believers, the cognitive element helps the injured person to understand the wrong, himself and the wrongdoer, the emotional element serves the person to overcome emotions resulting from the harm suffered and the person who caused harm, and the behavioural element helps to change behaviour; moreover, the religious element of forgiveness favours a religious person to understand God’s forgiveness of his own transgressions and wrongs done to others, and even negligence towards God, which is not experienced by a non-believer. God’s forgiveness given to the person’s own offenses and to others and Christ’s words “Father, forgive them, they do not know what they are doing” (Lk 23, 35) reactivates the believing person to forgive to himself and others, which is often useful in the process of development of own religiosity and personality.

According to the predictions of some researchers (Minow 1998; Zarzycka 2016), forgiveness should be seen as a psychological-spiritual process, which has a complex structure and is conditioned by many factors of personal and social nature, as well as religious nature; hence, we can talk about different types of forgiveness, which confirmed previous (Zarzycka 2016) as well as current research. It should be added that forgiveness, and its special component regarding the sense of forgiveness by God, is an important component of man’s religiousness. It is not always taken explicitly as a component of religiosity by researchers of the psychology of religion. The sense of God’s forgiveness is related to everyday decisions based on religion, of planning of the future and directing ones freedom towards God. And also the parameter of religious morality is a domain in which religious attitudes of morality, such as criteria of good or evil, religious arguments for fairness or unfairness of behaviours, and motives for taking action, are most important (Rydz et al. 2017).

This analysis has certain the limitations. Students participating in this study were recruited from one city, and from one university, and belong to one Roman Catholic Church. Only one measure of terminal values, of forgiveness, and of meaning in life was used, which limits the findings. There is a lack of a broader analysis of these received results—which stems from a shortage of previous research in this field among students who declare themselves believers and practitioners.

Despite that limitations described, that study results have several important implications. It is one of the first empirical studies that examined the relationship between terminal values, forgiveness taking into account the religious dimension, meaning in life in the lives of practicing and believing students in Poland. Research in the area of positive psychology has pointed the beneficial impact of forgiveness: forgiveness to self, forgiveness to others, and a sense of God’s forgiveness, as well as terminal values on meaning in life in the lives of practicing believer youth.

The analysis of the relationship between terminal values and forgiveness with meaning in life does not fully cover the issue raised. It may be a contribution to further more detailed research. In order to acquire knowledge in this area, research among other social groups as well as among other populations should be carried out.
